# Doctor performance assessment: development and impact of a new system

**DOI:** 10.1007/s40037-012-0009-0

**Published:** 2012-04-11

**Authors:** Karlijn Overeem

**Affiliations:** 1 IQ Healthcare, Radboud University Nijmegen Medical Centre, Nijmegen, the Netherlands; 2 Present Address: Department of Educational Research and Development, Faculty of Health, Medicine and Life Sciences, Maastricht University, PO Box 616, 6200 Maastricht, the Netherlands

**Keywords:** Multisource feedback, Medical specialists, Hospitals

## Abstract

Karlijn Overeem, general practitioner and researcher, defended her thesis on 15 November 2011. The thesis concerns the development and implementation of a performance assessment system for medical specialists (consultants) in Dutch hospitals. Besides the use of multisource feedback, the assessments consist of a portfolio and an assessment interview with a trained colleague. The thesis comprises: a review, two qualitative studies and three quantitative studies.

## Introduction

In the last 10 years, there is an increased emphasis on the assessment and improvement of doctors’ individual performance. This is necessary because doctors are confronted with knowledgeable patients, ever larger teams and exploding medical knowledge. This requires excellent communication, collaboration and management skills, which are all eminently suitable to assess and improve in daily practice. The current literature does not offer all insights necessary to understand how doctor performance assessment systems can contribute to improving doctor performance. Therefore, this thesis aims to study: how to design and implement a performance assessment system that is valid, reliable, feasible and effective in terms of improving doctor performance?

## Development and validation of a performance assessment system

In the first study we systematically reviewed the literature in order to provide an overview of methods and instruments available to assess doctor performance. We found six methods that can be used to assess doctor performance: video observation, simulated patients, direct observation, audit of medical records, multisource feedback (MSF) and portfolio. The methods observed varied greatly in feasibility, reliability, validity and effectiveness. MSF appeared to be most feasible in terms of time. On the basis of our review we designed a performance assessment system composed of:

1. MSF from colleagues (specialists within the same hospital), co-workers (nurses or other health care professionals) and patients;
2. Portfolio;
3. Assessment interview with a mentor;
4. A personal development plan.

Next, we studied the validity and reliability of three MSF measurement instruments, namely a colleague-completed instrument, a co-worker-completed instrument and a patient-completed instrument.

In this study with 150 specialists we found that the three MSF instruments were valid to evaluate specialists’ performance in the Netherlands. Our study revealed that five colleague evaluations, five co-worker evaluations and 11 patient evaluations are required to achieve reliable results.

## Impact of specialists’ performance assessments on performance improvement

In the third study, we evaluated the implementation of the assessment system in eight Dutch hospitals. The results demonstrated that specialists are significantly more satisfied with MSF containing narrative comments compared with MSF containing numerical scores. Furthermore, it became clear that the perceived impact of MSF that includes co-workers’ perspectives significantly exceeds the perceived impact of methods not including this perspective. Additionally, we interviewed 23 specialists to explore which factors are incentives, or disincentives, for specialists to implement suggestions for improvement from MSF. Specialists indicated that despite negative effects from contextual factors, such as high workload, the organisation of health care and public distrust, MSF can lead to progress when mentors stimulate reflection and concrete improvement goals. Regression analyses from our final study with 254 specialists revealed that indeed the quality of received mentoring and negative scores from colleagues were most influential on specialists’ reported change, explaining 34 % of variance. The thesis provides evidence on how we might optimise the role MSF can play in improving doctor performance. Mentoring and following up on improvement goals are essential for this.

